# A Compendium of Age-Related PheWAS and GWAS Traits for Human Genetic Association Studies, Their Networks and Genetic Correlations

**DOI:** 10.3389/fgene.2021.680560

**Published:** 2021-06-01

**Authors:** Seung-Soo Kim, Adam D. Hudgins, Brenda Gonzalez, Sofiya Milman, Nir Barzilai, Jan Vijg, Zhidong Tu, Yousin Suh

**Affiliations:** ^1^Department of Obstetrics and Gynecology, Columbia University Irving Medical Center, New York, NY, United States; ^2^Department of Genetics, Albert Einstein College of Medicine, Bronx, NY, United States; ^3^Institute for Aging Research, Department of Medicine and Genetics, Albert Einstein College of Medicine, Bronx, NY, United States; ^4^Department of Genetics and Genomic Sciences, Icahn School of Medicine at Mount Sinai, New York, NY, United States; ^5^Department of Genetics and Development, Columbia University Irving Medical Center, New York, NY, United States

**Keywords:** aging, age-related disease, age-related trait, biomarker, GWAS

## Abstract

The rich data from the genome-wide association studies (GWAS) and phenome-wide association studies (PheWAS) offer an unprecedented opportunity to identify the biological underpinnings of age-related disease (ARD) risk and multimorbidity. Surprisingly, however, a comprehensive list of ARDs remains unavailable due to the lack of a clear definition and selection criteria. We developed a method to identify ARDs and to provide a compendium of ARDs for genetic association studies. Querying 1,358 electronic medical record-derived traits, we first defined ARDs and age-related traits (ARTs) based on their prevalence profiles, requiring a unimodal distribution that shows an increasing prevalence after the age of 40 years, and which reaches a maximum peak at 60 years of age or later. As a result, we identified a list of 463 ARDs and ARTs in the GWAS and PheWAS catalogs. We next translated the ARDs and ARTs to their respective 276 Medical Subject Headings diseases and 45 anatomy terms. The most abundant disease categories are neoplasms (48 terms), cardiovascular diseases (44 terms), and nervous system diseases (27 terms). Employing data from a human symptoms-disease network, we found 6 symptom-shared disease groups, representing cancers, heart diseases, brain diseases, joint diseases, eye diseases, and mixed diseases. Lastly, by overlaying our ARD and ART list with genetic correlation data from the UK Biobank, we found 54 phenotypes in 2 clusters with high genetic correlations. Our compendium of ARD and ART is a highly useful resource, with broad applicability for studies of the genetics of aging, ARD, and multimorbidity.

## Introduction

In humans, physiological deterioration starts to occur at a young age (26–38 years) with loss of bone, cartilage, muscle mass and strength, and gain of abdominal fat (Belsky et al., 2015). Consequently, the incidence of age-related diseases (ARDs) increases exponentially with advancing age (Niccoli and Partridge, 2012). For example, the risk of developing Alzheimer’s disease doubles every 5 years after the age of 65 (Kaeberlein, 2013). Additionally, at least half of those individuals that reach 70 years of age suffer from 2 or more chronic diseases, a state known as multimorbidity (Barnett et al., 2012). However, an extended period of disease and dysfunction in late life is not an inevitable outcome, as studies on extremely long-lived individuals (i.e., centenarians), have found that they exhibit a significantly delayed age of onset of ARDs, resulting in a substantial compression of late-life morbidity (Partridge et al., 2018). This finding supports the recently widely embraced “geroscience hypothesis” (Kennedy et al., 2014), which posits that chronic diseases (i.e., ARDs) share a common underlying mechanism, the aging process itself, and that by targeting this process for intervention one can target multiple ARDs simultaneously. Thus, in order to uncover suitable targets for longevity interventions, it is important to comprehensively identify and characterize the relevant age-related traits (ARTs), including ARDs and their respective biomarkers. Once precisely defined, ARTs can then be used as proxy phenotypes of aging, providing a useful basis for both the quantification of the health status of aged cohorts (Fried et al., 2001; Mitnitski et al., 2001; Evert et al., 2003; Terry et al., 2008; Andersen et al., 2012; Belsky et al., 2015; Cieza et al., 2015; Caballero et al., 2017), as well as for studies that aim to identify the shared genetic architectures of ARDs and longevity (Belsky et al., 2015; Fortney et al., 2015; Johnson et al., 2015; Zenin et al., 2019; Melzer et al., 2020).

Although the common definition of an ARD is an increased rate of disease morbidity (i.e., incidence or prevalence) with age (Barnett et al., 2012), the specific criteria used to identify ARDs differs among studies. For example, Chang et al. (2019) identified 92 ARDs using a two-step linear regression framework and data from the Global Burden of Disease Study 2017 (GBD). Restricting their analysis to the adult population (25+ years), they used a linear model to test for whether the incidence rates of diseases increased with age. The authors additionally tested whether the incidence rates followed a convex relationship with age by way of a quadratic model. In another example, by utilizing self-reported disease data from the UK Biobank (UKBB), Donertas et al. (2020) generated age-of-onset profiles of common diseases (≥2,000 cases) and grouped the profiles into 4 clusters by using the partition around medoids algorithm. Out of 116 common diseases, the authors identified 25 ARDs which display a rapid increase in incidence after middle age (40+ years) and 51 ARDs that show a slow increase in incidence after early age (20+ years). However, the authors considered a limited age range, up to only 65 years old.

Human genetics research currently benefits from the wealth of publicly-available genetic association data compiled in the genome-wide association studies (GWAS; Buniello et al., 2019) and phenome-wide association studies (PheWAS) catalogs (Denny et al., 2010). However, for researchers of the genetics of aging and ARDs to fully take advantage of these highly valuable resources a comprehensive and well-defined list of ARTs is required, but to our knowledge, such a list has not yet been reported. Therefore, in this study, we aimed to identify a comprehensive list of ARTs, including ARDs and related biomarkers, using the whole traits and phenotypes present in the GWAS and PheWAS catalogs. As a result, we identified 463 ARTs, which we annotated with 100 international classification of diseases (ICD)-10 codes from the Gene ATLAS database. These traits map to 294 unique terms from Medical Subject Headings (MeSH) metadata, including 276 disease terms and 45 anatomy terms. Moreover, by overlaying our ARDs onto a human symptoms-disease network (HSDN), we identified 6 ARD subnetworks that represent disease groups with shared symptoms. Lastly, by translating our list of ARTs to UKBB phenotypes and clustering them by their genetic correlations, we found multiple ARTs with potentially shared genetic architectures. These results support the robustness of our methodology and the utility of the created resource for the field.

## Methods

### Definition of Age-Related Traits

**FIGURE 1:**
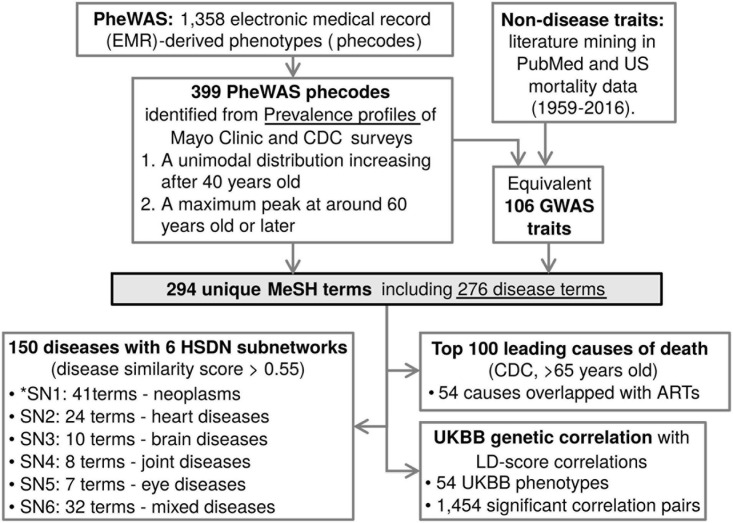
Identified age-related traits and diseases annotations for genetic resources. A schematic of the analysis flow of this study. From the PheWAS and GWAS catalogs, ARTs were identified, converted to Medical Subject Headings (MeSH) terms, and overlaid onto a human symptom-disease network (HSDN), top 100 leading causes of death, and UKBB genetic correlation data. *SN means subnetwork.

Google health cards^1^, which use data that is manually sourced from the Mayo Clinic and the Center for Disease Control and Prevention (CDC), were queried using 1,358 electronic medical record (EMR)-derived phenotypes (phecode) retrieved from the PheWAS catalog (accessed April 2017) to obtain prevalence profiles with age (Figure 1). TheWolfram| Alpha search engine^2^ was also used to compile “disease and patient-level statistics” data, sourced from CDC-conducted surveys, the National Ambulatory Medical Care Survey and the National Hospital Ambulatory Medical Care Survey, including the visitation data of 131,748 patients of United States healthcare providers from 2006 to 2007. From the disease prevalence profiles, ARTs were selected by the following criteria: (1) a unimodal distribution with prevalence increasing after mid-life (>40 years old); (2) a maximum peak at around 60 years old or later in the patient population size or prevalence rates. Non-disease traits such as biomarkers or endophenotypes associated with lifespan, aging, and ARTs were found and included through literature mining in PubMed, and mortality data for the United States (Vital Statistics NCHS’ Multiple Cause of Death Data, 1959–2016; http://www.nber.org/data/multicause.html). The ARTs were queried to find the equivalent traits/diseases from the GWAS and PheWAS catalogs.

### Annotation of International Classification of Diseases Codes and Gene Atlas

The GWAS Catalog database^3^ includes manually curated GWAS data, with >70,000 variant-trait associations from >3,500 publications (Buniello et al., 2019). As a reverse GWAS concept, the PheWAS catalog^4^ is a repository of phenome-wide association scans for associations between 3,144 single-nucleotide polymorphisms and 1,358 EMR-derived phenotypes (i.e., phecodes). This database is a powerful cross-validated source of replicated gene-phenotype associations from GWAS (Denny et al., 2010). The 399 phecodes of PheWAS ARDs were mapped to ICD-9^5^ (Denny et al., 2013) as well as ICD-10^6^ and ICD-10-CM^7^ (Wu et al., 2019). We merged the ICD codes to our ARD phecodes for external data annotation from the Gene ATLAS database. The Gene ATLAS database is a repository of genetic associations for 778 traits (self-reported or clinical diagnoses, tabulated as ICD-10 codes) that are found in at least 500 of the >452,000 UKBB participants (Canela-Xandri et al., 2018). Using the PheWAS ICD-10 codes, the equivalent ICD-10 codes or trait names from Gene ATLAS were annotated.

### Translation of Trait Terms to MeSH

The MeSH metadata^8^ provides a standardized vocabulary of medical terms with hierarchical categories, including biomedical information, including diseases, anatomy, chemicals and drugs, phenomena and processes, etc. (Lowe and Barnett, 1994). The 463 ARTs were queried to both the ‘‘Search’’^9^ and ‘‘MeSH on Demand’’^10^ functions on the MeSH webpage. The ART-MeSH term pairs are equivalent 1-to-1 matches with the following 4 non-equivalent pair instances; (1) some specific traits with no equivalent MeSH terms are mapped to parental MeSH terms (e.g., “Fracture of hand or wrist” mapped to “fracture” in MeSH terms); (2) some non-disease ARTs with no equivalent MeSH terms are alternatively mapped to MeSH anatomy, chemical, diagnosis, or phenomena terms (e.g., “Abnormal chest sounds” and “Stiffness of joint” mapped to “thorax” and “ankle joint” in MeSH terms, respectively); (3) co-morbidity traits with a common cause are mapped to the causal MeSH disease (e.g., “Hypertensive heart and/or renal disease” mapped to “hypertension” in MeSH terms); and (4) some ambiguous co-morbidity traits are mapped to one of those equivalent MeSH disease terms (e.g., “Cardiac arrest & ventricular fibrillation” mapped to “heart arrest” in MeSH terms). The ARTs were also independently mapped to relative MeSH anatomy terms by a 1-to-1 match. Some co-morbidity traits are mapped to one selected disease tissue (e.g., “cancer of kidney and urinary organs” mapped to “kidney” in MeSH terms).

Previously reported ARDs from Chang et al. (2019) and Donertas et al. (2020), as well as the top 100 leading causes of death according to data from the CDC^11^ were downloaded. These disease names were translated to equivalent MeSH terms in order to identify overlap with our list of 276 ARDs.

### Age-Related Disease Network

The HSDN data was extracted from the original study, which was constructed using data on 4,219 MeSH diseases, and their shared symptoms (Zhou et al., 2014). To identify the ARDs network of shared symptoms, the 276 MeSH diseases were overlapped with the HSDN. The disease similarity score criteria for the ARD network was empirically optimized as >0.55. The filtered ARD network is displayed in a yFiles Circular Layout with different colors for disease categories, unique node shapes for tissues/organs, and edge thickness for similarity scores by using Cytoscape software (Shannon et al., 2003).

### Annotation of UK Biobank Phenotypes and Identification of Genetic Correlations

Linkage disequilibrium (LD)-score correlations of 677 UKBB phenotypes were calculated and published electronically by the Neale lab^12^. In this study, the Neale lab’s UKBB genetic correlation data was downloaded (data accessed Dec. 29, 2020) and the UKBB phenotypes were mapped to the MeSH terms by equivalent 1-to-1 matches. The mapped MeSH terms were overlapped with the ART-derived MeSH terms to extract the age-related UKBB phenotypes. The raw *p*-values of genetic correlations were corrected to false discovery rate (FDR) by using the *p*.adjust function with method = “BH” option in R (Benjamini and Hochberg, 1995). To identify significant genetic correlations between the ARTs, a threshold of FDR < 0.05 was utilized. The genetic correlation *r*_*g*_ values were displayed as a heatmap by using the ComplexHeatmap library in R. To identify clusters of phenotypes, hierarchical clustering and the dynamic tree cut algorithm were applied by using hclust and cutreeDynamic functions (method = “tree” and cutHeight = 0.99 options) of dynamicTreeCut library in R.

## Results

### 276 Age-Related Diseases Across 45 Tissues Were Identified

In this study, we are using the terms ARDs to refer to diseases associated with age, and ARTs to refer to both ARDs and their biomarkers. To identify ARDs, first we obtained the prevalence profiles with age of 1,358 EMR-derived phecodes from the PheWAS catalog, and defined ARDs by these criteria; (1) a prevalence showing a unimodal distribution, increasing after mid-life (>40 years old); and (2) a maximum peak of prevalence occurring at around 60 years old or later. In addition to the PheWAS phecode-based diseases, non-disease traits were also manually identified through mining of PubMed literature and United States mortality data. ARTs corresponding to these phecodes and non-disease traits were then identified in the GWAS catalog. As a result, we found that 106 GWAS traits and 399 PheWAS traits (463 total traits) have age-associated prevalence profiles and are identified as ARTs (Supplementary Table 1).

**FIGURE 2:**
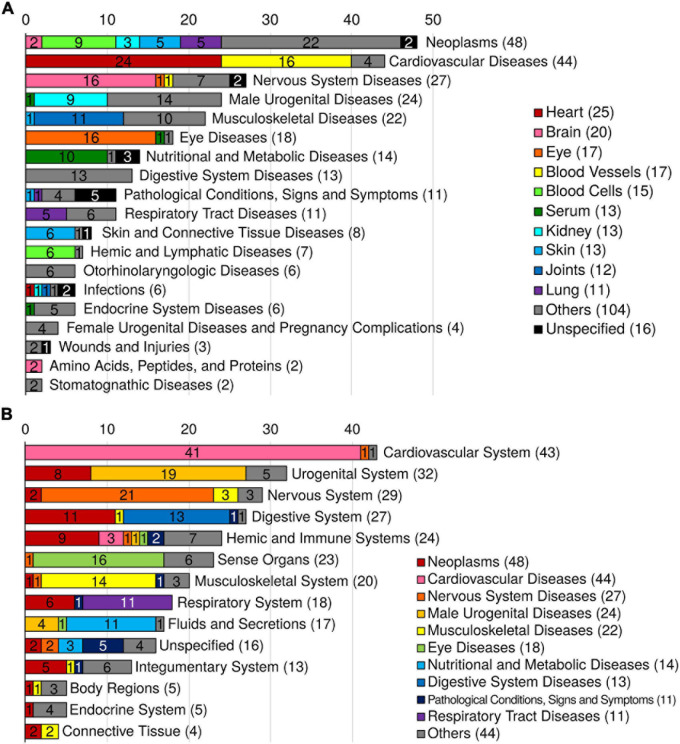
Summary of 276 MeSH disease and anatomy terms. **(A)** Distribution of the ARD MeSH disease categories and their corresponding tissues. The colors indicate the corresponding tissue. **(B)** Distribution of the ARD MeSH anatomy categories and their corresponding MeSH disease categories of ARDs. The colors indicate the corresponding MeSH disease category.

In many instances the GWAS and PheWAS catalogs use different terminologies for an equivalent disease trait (e.g., “Dementia” in the GWAS catalog and “Dementias” in the PheWAS catalog). To remove the redundancies and standardize the terms, we mapped the 463 ARTs to MeSH metadata and found 294 unique MeSH terms (Figure 1; see details in section “Methods”). The MeSH terms include 276 disease terms, 7 anatomy terms, 5 chemical terms, 3 diagnosis terms, 2 psychiatry terms, and a phenomena term. Of the 276 diseases, the top 5 most abundant disease categories were neoplasms (48 terms), cardiovascular diseases (44 terms), nervous system diseases (27 terms), male urogenital diseases (24 terms), and musculoskeletal diseases (22 terms; Figure 2A).

To identify the tissue distribution of the MeSH diseases (i.e., ARDs), we also mapped the 463 ARTs to MeSH anatomy terms. As a result, out of the total 45 tissues, the top 5 disease-specific tissues were heart (25 diseases), brain (20 diseases), eye (17 diseases), blood vessels (17 diseases), and blood cells (15 diseases; Figure 2A). And the top 5 MeSH anatomy categories were cardiovascular system (43 diseases), urogenital system (32 diseases), nervous system (29 diseases), digestive system (27 diseases), and sense organs (23 diseases; Figure 2B).

### Cancers and Diseases of the Heart, Brain, Joint, and Eye Are Grouped as Major Symptom-Shared ARDs

**FIGURE 3:**
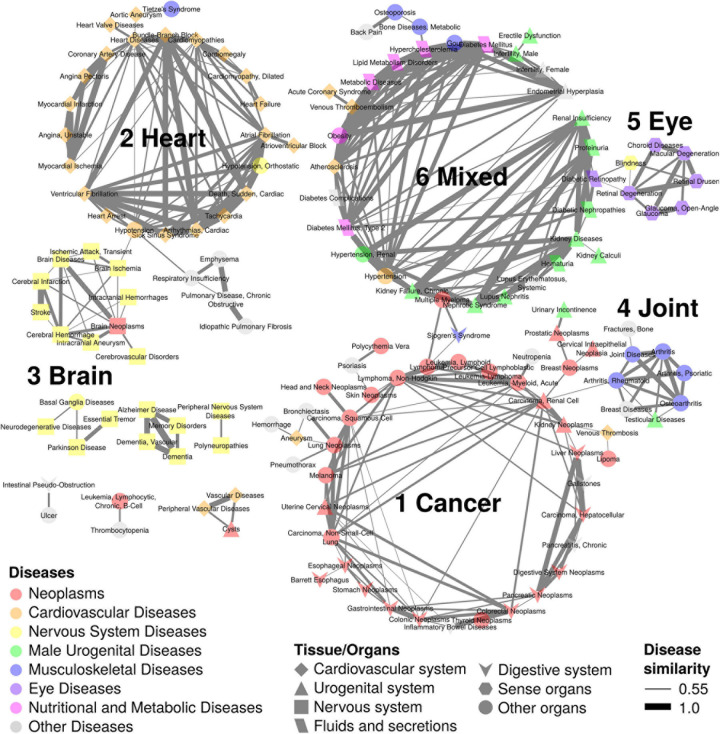
Disease network of 144 ARDs with 6 subnetworks. The ARD subnetworks of disease pairs with similarity scores > 0.55 are shown. There are 6 subnetworks; subnetwork 1 with 41 ARDs is composed of cancers; subnetwork 2 with 24 ARDs includes heart diseases; subnetwork 3 with 10 ARDs is composed of brain diseases; subnetwork 4 with 8 ARDs includes joint diseases; subnetwork 5 with 7 ARDs is composed of eye diseases; and subnetwork 6 with 32 ARDs contains mixed diseases. Node colors indicate different disease types. Node shapes represent different tissue/organs. Edge thickness indicates disease similarity score, with the thickest edge representing a disease similarity score = 1.

The HSDN was constructed from disease symptoms as well as shared genetic associations between diseases by calculation of disease similarity scores (Zhou et al., 2014). To identify the symptom-shared ARD groups, we overlapped the ARDs to the HSDN (Figure 1). Of the 276 diseases, we found that 144 ARDs are found in 6 primary subnetworks when using a disease similarity score criteria of >0.55 (Figure 3). We labeled the 6 subnetworks according to disease category and tissue type, with subnetworks 1–6 representing cancers, heart diseases, brain diseases, joint diseases, eye diseases, and various mixed diseases, respectively.

In subnetwork 1 (41 diseases), the biggest category of ARDs, there are the 30 neoplasm terms as well as 3 digestive system diseases (gallstones, inflammatory bowel diseases, and chronic pancreatitis), and 8 other diseases such as aneurysm and venous thrombosis (cardiovascular diseases), bronchiectasis and pneumothorax (respiratory tract diseases), neutropenia (hemic and lymphatic diseases), urinary incontinence (male urogenital diseases), psoriasis (skin and connective tissue diseases), and hemorrage (unspecified). The 30 neoplasm terms are distributed in various tissues such as gastrointestinal (5 terms; colonic, colorectal, stomach, digestive system, and gastrointestinal), blood (3 leukemia terms; precursor cell lymphoblastic leukemia-lymphoma, lymphoid leukemia, and acute myeloid leukemia; 2 lymphoma terms; lymphoma and non-Hodgkin lymphoma; a myeloproliferative neoplasm term, polycythemia vera), skin (3 terms; skin neoplasms, melanoma, and squamous cell carcinoma), esophagus (2 terms; esophageal neoplasms and Barrett esophagus), cervix (2 terms; cervical intraepithelial neoplasia and uterine cervical neoplasms), kidney (2 terms; renal cell carcinoma and kidney neoplasms), liver (2 terms; hepatocellular carcinoma and liver neoplasms), lung (2 terms; non-small-cell lung carcinoma and lung neoplasms), and other 6 sites (head and neck neoplasms, breast neoplasms, lipoma, pancreatic neoplasms, prostatic neoplasms, and thyroid neoplasms).

In subnetwork 2 (24 diseases), 20 heart disease terms can be further sub-divided into heart rhythm-related (7 terms; sick sinus syndrome, tachycardia, cardiac arrhythmias, atrial fibrillation, ventricular fibrillation, atrioventricular block, and bundle-branch block), myocardial ischemia-related (3 terms; angina pectoris, coronary artery disease, and myocardial ischemia), heart arrest (2 terms; cardiac sudden death and heart arrest), cardiomyopathy (2 terms; cardiomyopathies and dilated cardiomyopathy), and other 6 heart disease terms (heart diseases, myocardial infarction, unstable angina, cardiomegaly, heart failure, and heart valve diseases). Additionally, there are 2 blood disease terms (hypotension and orthostatic hypotension), a thorax disease term (Tietze’s Syndrome), and a blood vessel disease term (aortic aneurysm).

Brain diseases are found in subnetwork 3 (10 terms) including a brain cancer term (brain neoplasms), cerebral ischemia (2 terms; brain ischemia and transient ischemic attack), cerebral arteries occlusion (2 terms; cerebral infarction and cerebrovascular disorders), and other 5 brain disease terms (brain disease, intracranial hemorrhages, intracranial aneurysm, cerebral hemorrhage, and stroke). Joint diseases are clustered in subnetwork 4 (8 terms) such as arthritis, rheumatoid arthritis, joint diseases, and osteoarthritis as well as other 4 disease terms (testicular diseases, psoriatic arthritis, breast diseases, and bone fracture). Eye diseases are grouped in subnetwork 5, including glaucoma (2 terms; glaucoma and open-angle glaucoma), retina-related diseases (2 terms; retinal degeneration and retinal drusen), and 3 other eye diseases (choroid diseases, macular degeneration, and blindness).

In subnetwork 6 (32 terms), various diseases are clustered, including kidney diseases terms and related diseases (6 terms; kidney diseases, kidney calculi, chronic kidney failure, lupus nephritis, nephrotic syndrome, and renal insufficiency), male urogenital diseases (5 terms; renal hypertension, male infertility, erectile dysfunction, hematuria, and proteinuria), diabetes (2 terms; diabetes mellitus and type 2 diabetes mellitus) and diabetes complications (3 terms; diabetes complications, diabetic retinopathy, and diabetic nephropathies), nutritional and metabolic diseases (4 terms; hypercholesterolemia, lipid metabolism disorders, metabolic diseases, and obesity), cardiovascular diseases (4 terms; atherosclerosis, venous thromboembolism, hypertension, and acute coronary syndrome), musculoskeletal diseases (3 terms; metabolic bone diseases, osteoporosis, and gout), female urogenital diseases (2 terms; female infertility and endometrial hyperplasia), and 3 other diseases (e.g., multiple myeloma, back pain, and systemic lupus erythematosus).

### Half of the Top Causes of Mortality in the Aged Population Are ARDs

To evaluate the mortality of our ARDs, data on the top 100 clinical diagnoses (i.e., ICD-10 codes) of the leading cause of death for those over the age of 65 in the United States in 2018 was downloaded from the CDC, translated to MeSH terms, and overlapped with our 315 MeSH terms (Figure 1). As a result, 54 causes of death overlapped with our ARDs, including 18 cancers, 14 cardiovascular diseases, 10 nervous system diseases, 5 male urogenital diseases, 3 respiratory tract diseases, and 4 other diseases (gastrointestinal hemorrhage, myeloproliferative disorders, type 2 diabetes mellitus, and emphysema; “Rank_100” column in Supplementary Table 2). Intriguingly, the 54 high-mortality ARDs represent 85% of the total deaths from the top 100 causes of mortality.

### High Genetic Correlations Are Observed Between ARDs in Different Disease Symptom Subnetworks

**FIGURE 4:**
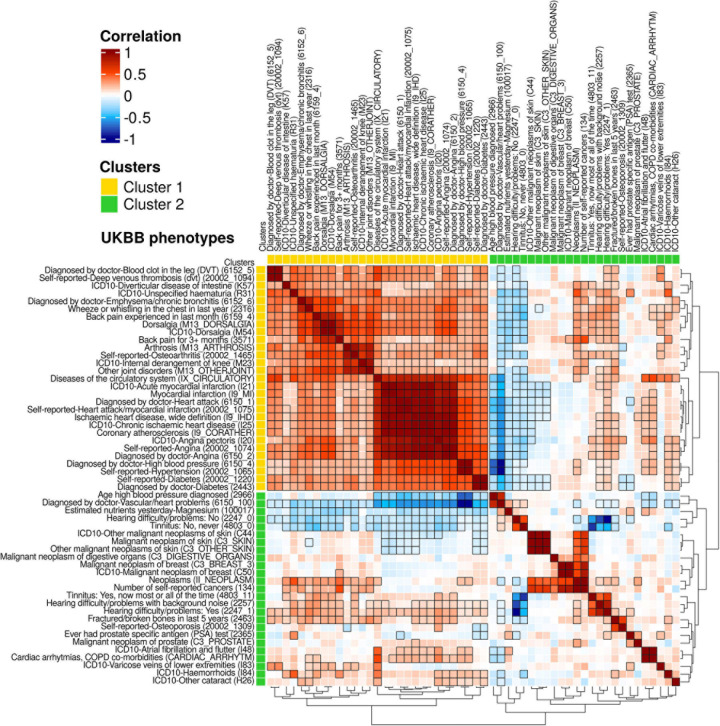
A heatmap for genetic correlations (*r*_*g*_) of 54 UKBB phenotypes. From the publicly available UKBB genetic correlation data (https://ukbb-rg.hail.is/rg_browser/), the genetic correlations (*r*_*g*_) of 54 UKBB phenotypes are displayed. By hierarchical clustering with the dynamic tree cut algorithm, 2 clusters of phenotypes were identified; Cluster 1 is composed of 17 phenotypes with positive correlations; cluster 2 includes 26 phenotypes with both positive and negative correlations. Unique ID of the UKBB phenotypes is noted in parentheses. Colors represent the values of genetic correlations (*r*_*g*_); dark red is *r*_*g*_ = 1, white is *r*_*g*_ = 0, and dark blue is *r*_*g*_ = −1. Significant genetic correlation pairs (FDR < 0.05) are indicated with black edged squares.

To identify shared genetic architectures that might exist between our ARTs, we downloaded genetic correlation data calculated between 677 UKBB phenotypes^13^ and translated these phenotypes to MeSH terms by equivalent 1-to-1 matches (Figure 1 and Supplementary Table 1). As a result, 54 UKBB phenotypes overlapped with 74 ARTs with 32 MeSH terms, and 1,454 pairs of phenotypes showed significant genetic correlation values (FDR < 0.05). By hierarchical clustering and using the dynamic tree cut algorithm, 29 and 25 phenotypes were grouped into 2 respective clusters (Figure 4).

The 29 phenotypes of cluster 1 have 746 significantly positive correlations (*r*_*g*_ > 0.11), with 2 main subclusters visible by examination of their tree structure. The first subcluster contains 14 phenotypes (*r*_*g*_ > 0.18) including 2 phenotypes of deep venous thrombosis (IDs: 6152_5 and 2002_1094), diverticular disease of intestine (K57), unspecified haematuria (R31), emphysema/chronic bronchitis (6152_6), wheeze or whistling in the chest in last year (2316), 4 phenotypes of backpain/dorsalgia (6159_4, M13_DORSALGIA, M54, and 3571), and 4 phenotypes of joint disorders (M13_ARTHROSIS, 20002_1465, M23, and M13_OTHERJOINT). The other subcluster shows positive correlations (*r*_*g*_ > 0.36) of 14 phenotypes than the first sub-cluster, of which, the 11 phenotypes are highly correlated (*r*_*g*_ > 0.71) including diseases of the circulatory system (IX_CIRCULATORY), heart attack/myocardial infarctions (4 phenotypes; I21, I9_MI, 6150_1, and 20002_1075), ischaemic heart diseases (2 phenotypes; I9_IHD and I25), coronary atherosclerosis (I9_CORATHER), and angina (3 phenotypes; I20, 20002_1074, and 6150_2). Hypertension (2 phenotypes; 6150_4 and 20002_1065) and diabetes (2 phenotypes; 20002_1220 and 2443) are also present in the second subcluster.

In cluster 2, 25 phenotypes have 114 significant correlation pairs. Of these 25 phenotypes, 8 cancer phenotypes (*r*_*g*_ > 0.38), including 3 skin cancers (C44, C3_SKIN, and C3_OTHER_SKIN), a digestive organ cancer (C3_DIGESTIVE_ORGANS), 2 breast cancers, C3_BREAST_3 and C50), and 2 other cancers (II_NEOPLASM and 134) appear subclustered from examination of the tree structure, as do 3 hearing loss-related phenotypes (*r*_*g*_ > 0.44; 4803_11, 2257, and 2247_1). The 4 phenotypes including vascular/heart problems diagnosed by doctor (6150_100), magnesium (100017), hearing difficulty/problems: No (2247_0), and tinnitus: No, never (4803_0) are negatively correlated (*r*_*g*_ < −0.08) with phenotypes in cluster 1. The latter 2 normal hearing phenotypes (2247_0 and 4803_0) also show negative correlations (*r*_*g*_ < −0.47) with the 3 hearing loss phenotypes. The phenotype age high blood pressure diagnosed (2966) and the 3 skin cancers (C44, C3_SKIN, and C3_OTHER_SKIN) displayed negative correlation (*r*_*g*_ < −0.10) with phenotypes in the second subcluster of cluster 1.

This result indicates that the ARDs grouped in different HSDN subnetworks (Figure 3) might share genetic associations. For example, venous thrombosis (subnetwork 1), myocardial infarction and angina pectoris (subnetwork 2), osteoarthritis and joint diseases (subnetwork 4), hematuria, back pain, hypertension, and diabetes mellitus (subnetwork 6) are included in cluster 1. For future analysis of genetic correlations, we additionally annotated 100 ICD-10 codes from the UKBB that have summary statistics stored in the Gene Atlas database (Canela-Xandri et al., 2018; Supplementary Table 1). In total, we annotated 144 UKBB phenotypes (including 93 ICD-10 codes) to 165 ARTs that have GWAS summary statistics either made available by the Neale lab^14^ or present in the Gene ATLAS database, or both.

## Discussion

In this study, we identified 463 ARTs in the GWAS and PheWAS catalogs. The disease prevalence profiles of these ARTs exhibit a unimodal distribution, increasing in prevalence after 40 years of age, and reaching a maximum peak at 60+ years of age. The 463 ARTs were annotated with clinical diagnosis code sets such as ICD-9, ICD-10, and ICD-10-CM from PheWAS data (Figure 1). To remove redundancies, the 463 ARTs were also translated to 294 unique MeSH terms, including 276 diseases. A combined analysis with both the MeSH disease terms and MeSH anatomy terms showed that the leading ARD categories are neoplasms (blood cells, skin, lung, kidney, and brain, etc.), cardiovascular diseases (heart, blood vessel, etc.), and nervous system diseases (brain, peripheral nerves, and spinal cord, etc.; Figure 2A). We also found that symptom-shared ARD subnetworks include cancers and diseases of the heart, brain, joint, eye, as well as others (Figure 3). Previously, we reported that shared biological pathways exist among the genes found to be associated with five major categories of ARDs using data from GWAS, including cancer, cardiovascular disease, neurodegenerative disease, metabolic disease, and other ARDs (Johnson et al., 2015). In this study, we report additional genetic correlations among ARDs in different subnetworks (Figure 4).

Since GWAS and PheWAS catalogs use different terminologies for the same trait (GWAS uses the experimental factor ontology and PheWAS uses ICD-9-CM billing codes), integrating data from these resources can prove difficult. Only 9% (42/463) of ART terms are shared between both resources, which would lead to high redundancy in any list of ARTs that relied on simple integration of the two datasets. Our solution was to use the standardized terminology provided by MeSH metadata, which allowed for the creation of a non-redundant set of ARTs, and also allowed us to expand our annotation to other resources, such as the HSDN and UKBB. However, 1-to-1 matching from traits to MeSH terms can present a separate set of difficulties, such as traits with no equivalent MeSH terms and co-morbidity traits that share a common cause. These issues can be partially circumvented by using alternative terms, such as parental terms, alternative category terms, or causal terms. However, ambiguous co-morbidity terms (e.g., “Cancer of kidney and urinary organs”) has to be translated to only one MeSH term (e.g., “Kidney Neoplasms”), which then leads to a biased distribution of diseases and tissues. For future studies, the development of a hub for multiple clinical-genomic resources which uses standardized terms (i.e., MeSH or ICD codes) should be prioritized and would be a great benefit for the field.

Recently, Donertas et al. (2020) and Chang et al. (2019) reported 76 ARDs, using UKBB data, and 92 ARDs, using GBD data, respectively. Compared with our list of ARDs, 61% of the diseases (46/76 diseases) and 49% (45/92 diseases) overlap (Supplementary Table 2). Surprisingly, only 7 MeSH diseases are shared between all 3 lists of ARDs, including 4 cardiovascular disease terms (hypertension, cardiovascular diseases, cardiac arrhythmias, and heart valve diseases), 2 eye disease terms (cataract and glaucoma), and stroke. These results suggest that there is a need to develop a consensus with regard to defining what constitutes an ARD, one that can be universally applied to different populations. In this study, by starting with EMR-derived phecodes in the PheWAS catalog and extensive text mining, the largest number of traits considered thus far, we were able to provide a comprehensive list of ARDs and ARTs available in the GWAS and PheWAS databases. When combined with population-scale clinical diagnosis data, for example those of the UKBB, our list can help identify shared genetic mechanisms of co- or multi-morbidity in the elderly. Furthermore, our tissue-specific ARD list can be useful in the investigation of the underlying tissue-specific mechanisms of aging, and can also be used as proxy phenotypes of aging.

Major ARDs (e.g., cancer, cardiovascular disease, dementia, hypertension, osteoporosis, and stroke) show rates of mortality and morbidity that are strongly associated with age (Andersen et al., 2012). Indeed, our study showed that out of the top 100 leading causes of death for individuals over the age of 65, 54 diseases are present in our list of ARDs and account for 85% of total deaths. This result indicates that our ARDs might be useful as proxy phenotypes of life expectancy.

In this study, we provide a resource for the fields of aging and ARD genetics, identifying 499 ARTs, and reporting their respective ICD codes, MeSH terms, and symptom similarity networks, as well as their genetic correlations. The data sets generated by our study represent important, but currently lacking, resources for the aging research community.

## Data Availability Statement

Publicly available datasets were analyzed in this study. This data can be found here: http://www.nber.org/data/multicause.html, https://www.ebi.ac.uk/gwas/home, https://phewascatalog.org, https://phewascatalog.org/phecodes, phewascatalog.org/phecod es_icd10, https://phewascatalog.org/phecodes_icd10cm, gene atlas.roslin.ed.ac.uk/, https://meshb.nlm.nih.gov/, meshb.nlm. nih.gov/search, meshb.nlm.nih.gov/MeSHonDemand, won der.cdc.gov/ucd-icd10.html, and https://ukbb-rg.hail.is/rg_browser/.

## Author Contributions

YS designed and instructed the study. S-SK analyzed the results and wrote the first draft of the manuscript. AH provided useful suggestions in methodology and wrote the first draft. BG generated the raw data. SM, NB, JV, and ZT provided useful suggestions in methodology. All authors contributed to the article and approved the submitted final manuscript.

## Conflict of Interest

The authors declare that the research was conducted in the absence of any commercial or financial relationships that could be construed as a potential conflict of interest. The handling editor declared a past co-authorship, with one of the author, NB.

## Footnotes

1 https://www.google.com, accessed September 2017.

2 https://www.wolframalpha.com, accessed October 2017.

3 https://www.ebi.ac.uk/gwas/home, accessed June 2020.

4 https://phewascatalog.org

5 https://phewascatalog.org/phecodes

6 https://phewascatalog.org/phecodes_icd10

7 https://phewascatalog.org/phecodes_icd10cm

8 https://meshb.nlm.nih.gov/

9 https://meshb.nlm.nih.gov/search

10 https://meshb.nlm.nih.gov/MeSHonDemand

11 http://wonder.cdc.gov/ucd-icd10.html

12 https://ukbb-rg.hail.is

13 https://ukbb-rg.hail.is/rg_browser/

14 https://github.com/Nealelab/UK_Biobank_GWAS
